# Toward a New Definition of “Healthy” Food: Issues and Challenges

**DOI:** 10.1016/j.cdnut.2023.100080

**Published:** 2023-04-19

**Authors:** Kimberly Siu, Adam Drewnowski

**Affiliations:** 1 Nutritional Sciences Program, School of Public Health, University of Washington, Seattle, WA, United States; 2 Center for Public Health Nutrition, University of Washington, Seattle, WA, United States

**Keywords:** Food and Drug Administration, proposed regulation, healthy, Food and Nutrient Composition Database for Dietary Studies FNDDS 2017-18, nutrients, food groups

## Abstract

Dietary Guidelines for Americans (DGA) have become increasingly food-based guidelines. The Healthy United States–Style Eating Pattern features fruits, vegetables, whole grains, and low-fat dairy, with limits placed on added sugar, sodium, and saturated fat. Recent measures of nutrient density have followed suit, incorporating both nutrients and food groups. Most recently, the United States Food and Drug Administration (FDA) has proposed to redefine the concept of a “healthy” food for regulatory purposes. To qualify as healthy, foods will need to contain specific minimum amounts of fruits, vegetables, dairy, and whole grains, with limits placed on added sugar, sodium, and saturated fat. The present concern was that the proposed criteria, formulated by the FDA per Reference Amount Customarily Consumed, were so stringent that few foods would pass. We applied the proposed FDA criteria to foods in the USDA Food and Nutrient Database for Dietary Studies (FNDDS 2017–2018). The criteria were met by 58% of fruits, 35% of vegetables, 8% of milk and dairy products, and 4% of grain products. Many foods commonly considered to be healthy by consumers and the USDA alike did not pass the proposed FDA criteria. Federal agencies seem to define healthy in different ways. Our findings have implications for the formulation of regulatory and public health policies. We recommend that nutrition scientists be involved in the development of federal regulations and policies that affect American consumers and the food industry.

## Introduction

The Healthy United States–Style Eating Pattern was developed by the United States Department of Agriculture (USDA) to show that Dietary Guidelines for Americans (DGA) could be met in a variety of ways [1]. The main types of healthy foods in the Healthy United States–Style Eating Pattern were vegetables, fruits, whole grains, low-fat dairy, poultry, and seafood, along with eggs, nuts, seeds, and soy products [1]. The definition of what foods are “healthy” has always oscillated between nutrients and food groups [2], with food groups currently in the ascendant [3]. A new hybrid nutrient density score, NRFh4:3:3, awarded higher scores to foods that included fruit, whole grains, and dairy and contained no added sugar, sodium, or saturated fat [3].

Most recently, the FDA has proposed an updated definition of healthy to better reflect current nutrition science and federal dietary guidelines [4]. Foods would qualify as healthy if they contained minimum amounts of fruits, vegetables, whole grains, and dairy, with strict limits placed on added sugar, sodium, and saturated fat [4]. All calculations were per reference amount customarily consumed (RACC) [4].

This new thinking may have arisen from the 2015 KIND petition to the FDA [5], which argued that some foods generally perceived to be healthy (nuts, avocado, and salmon) would be disqualified on the basis of their nutrient content because they contained saturated fat. The argument was to redefine healthy based on food groups [5]. The present concern was that the FDA-proposed criteria, this time based on both nutrients and food groups, were so stringent that few foods would pass. In that case, foods generally perceived to be healthy (whole grains, vegetables, and yogurt) would be still be disqualified on the basis of their food group and their nutrient content [6].

We put the proposed FDA definition of healthy to the test. First, we merged the USDA Food and Nutrient Database for Dietary Studies (FNDDS 2017–2018) with the USDA Food Patterns Equivalents Database (FPED) [7,8]. Then, we applied the proposed FDA criteria to fruit, vegetable, milk and dairy, and grain groups following the USDA coding system. Excluded from analyses (for now) were snacks and sweets, mixed dishes, and protein foods.

## Materials and Methods

The proposed FDA standards were based on both the nutrients and food groups. The adequacy criteria were set at ½-cup (118.3 mL) equivalent per RACC for fruits and vegetables and ¾-cup (177.4 mL) equivalent per RACC for milk and dairy products. Grain products had to contain a minimum of ¾-oz (21.3 g) equivalent of whole grains per RACC. Raw, whole fruits and vegetables were automatically healthy by default. Maximum allowed amounts of added sugar, saturated fat, and sodium were expressed as percentage daily values (%DVs). For added sugar, the standard was set at 0 %DV (i.e., 0 g per RACC) for fruits and vegetables and at ≤5 %DV (assumed to be 2.5 g per RACC) for milk and dairy and for grains. For saturated fat, the standard was set at ≤5 %DV (assumed to be ≤1 g per RACC) for fruits, vegetables, and grain products but was raised to ≤10 %DV (<2 g per RACC) for milk and dairy. The limit on sodium was ≤10 %DV (assumed to be ≤230 mg per RACC) for all food groups. The proposed criteria are shown in Table 1.

**TABLE 1:** The FDA-proposed criteria for fruits, vegetables, milk and dairy, and grains per RACC [4]

Food group	Food group equivalent minimum	Added sugar limit (%DV)	Sodium limit (%DV)	Saturated fat limit (%DV)
Fruits	≥½ cup-eq fruit	≤0	≤10	≤5
Vegetables	≥½ cup-eq vegetable	≤0	≤10	≤5
Milk and dairy	≥¾ cup-eq dairy	≤5	≤10	≤10
Grains	≥¾ oz-eq whole grain	≤5	≤10	≤5

%DV = percentage daily value; RACC, reference amount customarily consumed.

The USDA FNDDS 2017–2018 is a part of the What We Eat in America (WWEIA) dietary component of the National Health and Nutrition Examination Surveys [7]. The 2017–2018 FNDDS contains 7083 foods and beverages, aggregated into 16 food groups, 49 food subgroups, and 167 food categories. We merged the FNDDS 2017–2018 with the USDA FPED, which converts each food or beverage into 37 USDA Food Pattern components [8]. Both databases can be downloaded from the USDA FoodData Central [9]. The reason for using the FNDDS 2017–2018 was that it contains nutrient data for foods as consumed, aligning with American eating habits and consumption patterns for healthy and unhealthy foods.

The WWEIA food groups of interest were as follows: *1*)fruits; *2*) vegetables, including starchy vegetables and potatoes; *3*) milk and dairy, including milk, flavored milk, yogurt, and cheese; *4*) grains, including breads, rolls, and tortillas; cooked cereals; cooked grains; quick breads and bread products; and ready-to-eat cereals. RACC values were obtained from the FDA and were manually attached to FNDDS food categories and item codes [10]. All RACC values were in grams. Beverages with RACC values in milliliters were converted to grams (1 g = 1 mL). Foods that could not be matched with RACC values were excluded. Statistical analyses used Microsoft Excel (version 16.66; Microsoft Corporation) and R (version 2022.07.1; The R Foundation for Statistical Computing).

## Results

Fruits and vegetables in the WWEIA studies can be fresh, frozen, canned, or further processed. Only raw, unprocessed fruits and vegetables automatically qualified as healthy. Table 2 demonstrates that of the 118 fruits, only 36 were raw. Altogether, 49 fruit items (42%) failed the proposed criteria. The main reasons for failure were insufficient content (≥½ cup equivalent) of fruit per RACC (34 items or 41%) or too added sugar (40 items or 49%). The only healthy canned fruits were juice pack oranges, fruit cocktail, peaches, pears, and pineapples.

**TABLE 2:** Food items passing and failing the FDA “healthy” criteria by food group∗

USDA food group	Fruits	Vegetables	Milk and dairy	Grains
*N*	118	627	239	624
Healthy	69 (58)	217 (35)	20 (8)	22 (4)
Not Healthy	49 (42)	410 (65)	219 (92)	602 (96)
Food items failing each FDA healthy criteria				
Not raw, whole	82 (69)	565 (90)	—	—
Insufficient FGE per RACC^1^	34 (41)	79 (14)	125 (52)	466 (75)
Excess added sugar^1^	40 (49)	40 (7)	101 (42)	284 (46)
Excess sodium^1^	1 (1)	230 (41)	49 (21)	123 (55)
Excess saturated fat^1^	13 (16)	316 (56)	123 (51)	154 (25)

FGE, food group equivalent; RACC, reference amount customarily consumed.

1 Only those fruit and vegetable products that were not raw and whole were screened for FGE and added sugar, sodium, and saturated fat. Those calculations were based on 82 fruit and 565 vegetable items.

∗ Values are presented as *n* (%).

Of the 627 vegetables, only 62 (10%) were raw and, therefore, healthy by default—with another 155 (27%) meeting all criteria. The rest failed. The main reasons for failure were excessive amounts of sodium (230 items or 41%) and/or insufficient content (≥½ cup equivalent) of vegetables per RACC (79 items or 14%). It is well established that vegetables as cooked and consumed are a major source of sodium in the American diet [11]. Failing the FDA criteria were cooked fresh carrots, kale, beets, spinach, and tomatoes—all of which contained too much sodium. Most reduced sodium-canned vegetables passed. White potatoes (baked/boiled/roasted/stewed) passed, although other studies questioned their nutritional value [12]. Canned collards, greens, kale, mustard greens, pumpkin, blackened peas, lima beans, and sweet potatoes failed. The only healthy French fries product was school potato tots.

Of a lot of milk and dairy products, only 20 (8%) met the healthy criteria. Those that failed had insufficient content (≥¾ cup equivalent) of dairy per RACC (125 items or 52%), too much saturated fat (123 items or 51%), too much added sugar (101 items or 42%), and/or too much sodium (49 items or 21%). Low-fat and skim milk passed. Most yogurts failed because they contained less than ¾-cup equivalent dairy per RACC. The only healthy cheeses were nonfat or fat-free Mozzarella cheese and reduced fat Swiss cheese. By contrast, chocolate milk made from sugar-free dry mix with low-fat (1%) and fat-free (skim) milk passed.

The situation was even starker for the grains. Of the 624 grain products, only 22 (4%) were healthy. Those that failed had insufficient content (≥¾ oz equivalent) of whole grains per RACC (466 items or 75%), too much sodium (342 items or 55%), too much added sugar (284 items or 46%), and too much saturated fat (154 items or 25%). General Mills Cheerios and Fiber One qualified as healthy, along with select oat cereals, shredded wheat, and muesli. Oatmeal failed because of insufficient amounts (¾ oz equivalent) of whole grain per RACC. Very few breads were passed. Among the breads that did pass were whole or cracked wheat breads, gluten-free bread, high-protein bread, pita bread, rice bread, and Ethiopian bread. Among the cooked grains, only quinoa and buckwheat groats with no added fat passed. All types of rice, pasta, noodles, tortillas, rolls, breadsticks, bagels, and muffins failed.

## Discussion

These analytical results call into question the proposed regulations that are so stringent that they cannot be met by most foods in the current marketplace. Although the definition of healthy needs to be science-based, it also needs to be sufficiently feasible to be implemented through dietary education and guidance. Our analysis of the proposed FDA regulation has clearly identified an underlying problem. All too often, the definition of healthy is purely arbitrary, and standards used by one federal agency are very different from standards used by another. To complicate matters, the definition of healthy has long been linked to the absence of problematic nutrients (added sugar, sodium, and saturated fat) as opposed to any beneficial nutrients that a food might actually contain [5]. The DGA 2020-25 identified sparkling water as a “nutrient-dense” food [1].

This places consumers in a difficult position. Although the USDA DGA promote the consumption of healthy foods [1], the proposed FDA regulation seems to imply that few such foods exist. This may discourage consumers from following dietary guidance; it may also discourage the food industry from pursuing product reformulation to meet the new criteria. If only 4% of grain products will qualify as healthy, why bother?

The present recommendation is for the FDA to delay the final ruling and focus on clarifying some methodology issues. One issue is the reference amount or portion size. Although the USDA DGA and the FPED use ounce equivalent and cup equivalent, the FDA uses RACC values that are expressed in grams, but not always. By contrast, saturated fat, added sugar, and sodium content of foods are expressed per 100 g. The lack of crosswalks among the databases has given rise to some mathematical impossibilities. For example, the USDA defines 1.5 oz (42.5 g) of cheese as 1 cup equivalent of dairy. The FDA proposal is for a minimum of ¾-cup equivalent of dairy (31.9 g by our calculations) per RACC, but the RACC for cheese is 30 g. It is impossible to squeeze 31.5 g of cheese into 30 g, so cheese failed as did most yogurts. Because RACC values are highly variable, the same problem may apply to other food groups. It would be simpler to base calculations per 100 g.

Second, when deciding whether a food is healthy, it is useful to consider how it is consumed by most people. Less than 10% of vegetables in the FNDDS were described as raw. The FDA-proposed regulation was silent on sliced carrots and dried, cut, frozen, and puréed vegetables that were not specifically included in the exemption. Furthermore, most vegetables are eaten with fat and some salt, as reflected in the WWEIA studies. It may not have been the FDA intent, but 65% of the vegetables failed.

Third, the fact that so few grain products passed the proposed criteria ought to be a matter of public health concern. Based on information obtained from FPED, most grain products, such as oatmeal and brown rice, did not contain a minimum of ¾-oz equivalent of whole grains per RACC [13]. The proposed regulation may have overemphasized the importance of whole grains, without considering the multitude of nutrients that grain products provide [14].

In summary, many foods commonly perceived to be healthy failed to meet the proposed FDA criteria. As this analysis show, fruits, vegetables, dairy, and grain products struggled. Oatmeal, yogurt, brown rice, and many vegetables failed. This was precisely the problem that the new definition was meant to remedy.

However, this analysis has limitations. First, in the absence of database crosswalks, RACC values had to be manually attached to FNDDS food codes, allowing for errors. The FDA lists RACC values for food groups rather than for FNDDS codes, allowing for misinterpretations. Second, although RACC values were checked by 2 investigators, the lack of automation in this process and the lack of standardization of RACC values leave room for more errors. Although USDA portion sizes were in cup equivalent and ounce equivalent, calculations of %DVs based on 100 g complicated further. With a multitude of units used by the USDA and the FDA (i.e., cup equivalent, ounce equivalent, grams, milliliters, and RACC values) and absence of linkages across databases, this analysis represent the best effort to assess the FDA-proposed definition for healthy. It would be helpful for the USDA and the FDA to jointly attach RACC values to every food item in the FNDDS.

The proposed regulations may pose a problem for the design and implementation of the DGA 2025-2030. Although the USDA DGA promote the consumption of healthy foods [1], the proposed FDA regulation suggests that such foods are rare. However, the proposed FDA definition of healthy may not take effect until 2029, giving consumers—and the food industry—some time to consider the proposal in detail and to conduct further analyses. The proposed criteria severely limit the number and types of fruits, vegetables, dairy, and grain products that will be able to bear the “healthy” claim.

The present recommendation is for nutrition scientists to become more engaged in shaping future regulatory policies, if only to ensure that federal regulations align with public health goals. Analyses of publicly available databases, such as those ones conducted in this study, ought to be a key component of feasibility assessments of proposed federal regulations.

## Funding

This research was supported by the Top Scholar Award to KS from the University of Washington Graduate School and School of Public Health Nutritional Sciences Program.

## Author disclosures

AD is the developer of the Nutrient Rich Food (NRF) index, a nutrient profiling model; is a member of the Nestle scientific advisory board, a member of the FrieslandCampina expert panel, and invited member of the Carbohydrate Quality Coalition funded by Potatoes USA; and has received grants, contracts, and honoraria from entities, both public and private, with an interest in nutrient profiling methods, and nutrient density of foods, meals, and total diet. KS has no conflicts to disclose.

## Author contributions

The authors responsibilities were as follows—KS: performed the analyses and took the lead in the first draft with contributions from AD; both authors revised, reviewed, and commented on subsequent drafts of the manuscript; both authors read and approved the final manuscript.

## Data availability

The data described in the manuscript are freely available without restriction and can be downloaded from the USDA FoodData Central. https://fdc.nal.usda.gov/fdc-app.html#/.
